# Calcium signalling in mammalian cell lines expressing wild type and mutant human α1-Antitrypsin

**DOI:** 10.1038/s41598-019-53535-1

**Published:** 2019-11-21

**Authors:** Nancy T. Malintan, Steven D. Buckingham, David A. Lomas, David B. Sattelle

**Affiliations:** 1 Centre for Respiratory Biology, UCL Respiratory, Rayne Building, University College London, 5 University Street, London, WC1E 6JF UK; 2 UCL Queen Square Institute of Neurology, London, WC1N 3BG UK

**Keywords:** Biological techniques, Medical research

## Abstract

A possible role for calcium signalling in the autosomal dominant form of dementia, familial encephalopathy with neuroserpin inclusion bodies (FENIB), has been proposed, which may point towards a mechanism by which cells could sense and respond to the accumulation of mutant serpin polymers in the endoplasmic reticulum (ER). We therefore explored possible defects in Ca^2+^-signalling, which may contribute to the pathology associated with another serpinopathy, α_1_-antitrypsin (AAT) deficiency. Using CHO K1 cell lines stably expressing a wild type human AAT (MAAT) and a disease-causing polymer-forming variant (ZAAT) and the truncated variant (NHK AAT), we measured basal intracellular free Ca^2+^, its responses to thapsigargin (TG), an ER Ca^2+^-ATPase blocker, and store-operated Ca^2+^-entry (SOCE). Our fura2 based Ca^2+^ measurements detected no differences between these 3 parameters in cell lines expressing MAAT and cell lines expressing ZAAT and NHK AAT mutants. Thus, in our cell-based models of α1-antitrypsin (AAT) deficiency, unlike the case for FENIB, we were unable to detect defects in calcium signalling.

## Introduction

Alpha-1 Antitrypsin (α_1_-antitrypsin, AAT) is a member of the serpin family of protease inhibitors^1–3^. The primary source of systemic AAT is the liver^4–6^ from which it is discharged into the circulation where it plays a protective role by reducing the impact on cells and tissues of the enzymatic activity of inflammatory cells^7–9^. For example, if AAT is absent or defective as a result of mutations, neutrophil elastase breakdown of elastin is unregulated, thereby compromising lung elasticity^10^. This results in emphysema or chronic obstructive pulmonary disease (COPD)^11,12^ and liver cirrhosis^13,14^ in adults or children. AAT is one of several serpinopathies for which drug treatment is poor or completely lacking^15–17^.

The Z variant, a point mutation resulting from a glutamate to lysine switch at position 342 (E342K), is the most common AAT mutation (ZAAT) resulting in AAT deficiency^3,18–20^. The ZAAT mutation affects the folding integrity of the protein^21^ resulting in the generation of ordered protein polymers that are not secreted and instead are retained in the endoplasmic reticulum (ER) of hepatocytes^22–25^. Accumulated ZAAT polymers lead to liver disease via a toxic gain of function^20,26^. In contrast, the severe impact detected in the lungs of ZAAT individuals is due to a damaging loss of AAT function^7^, which stems from low levels of circulating AAT^27,28^. Under normal conditions, cells clear misfolded proteins from the ER by means of the unfolded protein response (UPR)^29–31^, thereby maintaining protein homeostasis^32,33^. However, in cells harbouring ZAAT polymers, the UPR is not activated despite the misfolding of ZAAT. Instead, the ER overload response is triggered^25,34^, ultimately leading to ER stress. Such ER swelling has been detected in both the ZAAT polymer-forming mutant cells and NHK-AAT cells containing a truncated AAT mutant in which the protein is degraded but not in the wild type (MAAT) cells^25^.

The versatility and plasticity of the ER enable it to respond to many stimuli and challenges^32,35,36^. Thus, ER remodelling could be one of the cell’s mechanisms to alleviate ER stress^36^. Although numerous studies have explored AAT deficiency in health and disease^7,37–39^, the pathological mechanism(s) by which ZAAT polymers in AAT deficiency cause cellular damage is poorly understood^4,20,40,41^.

Cellular processes are intricate and dynamic^42^, and intracellular calcium (Ca^2+^) controls many physiological processes via its actions as a second messenger^43–48^. Maintenance of intracellular calcium levels is crucial for cell health^46,48–50^. Many human cardiac and neurological diseases result from disturbance in intracellular Ca^2+^ balance^51,52^ due to defective components of the Ca^2+^ signaling machinery^53–56^. While the signalling role of Ca^2+^ in the cytosol is well known, it may also participate in signalling within the ER^44,57–59^. In 2009 Davies *et al*. suggested a possible role for calcium signalling in a serpinopathy, the autosomal dominant form of dementia familial encephalopathy with neuroserpin inclusion bodies (FENIB)^60^. In a PC12 cells model of the disease, polymers of mutant neuroserpin accumulate within the ER^60^ and activate nuclear factor kappa B (NF-κB) via a pathway independent of the IRE1, ATF6, and PERK branches of the canonical UPR. Studies with thapsigargin (TG), an ER Ca^2+^-ATPase blocker, pointed to a role for calcium signalling in FENIB^60^.

To explore whether or not Ca^2+^-signalling components are associated with another type of serpinopathy; AAT deficiency, we measured basal free calcium, responses to TG, and store-operated Ca^2+^-entry (SOCE) in CHO K1 cell lines stably expressing the wild type human AAT (MAAT) and the disease-causing variants (ZAAT and NHK AAT).

## Materials and Methods

### Cell lines

Alpha1-antitrypsin (AAT) Tetracycline-inducible (Tet-ON) CHO-K1 stable cell lines expressing the human wild type AAT (MAAT), polymerogenic AAT E342K (ZAAT) and a truncated AAT variant L318fsX17 (NHK AAT) were generated and maintained as previously described^25^. The cells were maintained in DMEM (Sigma-Aldrich) supplemented with 10% v/v Tet-free FBS (Takara Bio Europe, Clontech Inc.), 1% v/v MEM non-essential amino acids (Life Technologies), 1% v/v penicillin-streptomycin 10,000 U/ml (Life Technologies), 200 μg/ml Geneticin (Life Technologies) and 200 μg/ml hygromycin B (Life Technologies) at 37 °C and 5% CO_2_. A non-AAT expressing Tet-ON CHO-K1 parental cell line was used as a control and maintained in DMEM as outlined above but without hygromycin B.

### Antibodies

Monoclonal mouse anti-total antitrypsin 2G7 (1:100 v/v)^61^ was used to detect both monomeric and polymer forms of AAT. Monoclonal mouse anti-polymer 2C1 antibody (1:25 v/v, culture media supernatant)^62^ recognised the AAT polymer. Polyclonal rabbit anti-GAPDH (#ab9485, 1:5,000 v/v) and rabbit anti-Giantin (#ab24586, 1:100 v/v) antibodies were obtained from Abcam. Polyclonal rabbit anti-Calreticulin (PA3–900, 1:500 v/v) was obtained from Thermo Scientific. The secondary antibodies donkey anti-mouse Alexa Fluor 555 and donkey anti-rabbit Alexa Fluor 488 (1:1,000 v/v) used in immunocytochemistry were obtained from Molecular Probes, Invitrogen. Goat anti-mouse IRDye 680 and anti-rabbit IRDye 800 infrared secondary antibodies used for western blotting (1:20,000 v/v) were purchased from LI-COR Biosciences.

### Cell preparation for microscopy imaging

Control and AAT-Tet ON CHO K1 cells expressing mutants were allowed to adhere overnight to sterilised poly-D-lysine (Sigma Aldrich) coated (0.1 mg/ml) 22 mm diameter glass coverslips, (No. 1.5, VWR International) in 35 mm wells within 6-well cell culture plates (Techno Plastic Products AG). The AAT-Tet ON CHO K1 expressing cells were induced to express AAT by adding doxycycline (Takara Bio Europe, Clontech Inc.) to a final concentration of 1 μg/ml 48 h before processing for western blotting, immunocytochemistry or calcium imaging.

### Western blotting analysis

Cells were harvested by scraping, then homogenised and solubilised in buffer containing 25 mM Tris-HCl (pH8.0), 150 mM NaCl, 1% Nonidet-P40 (Roche Diagnostic) and complete protease inhibitors-EDTA free (Roche Diagnostic). Total protein concentrations were determined by colourimetry using Bradford reagent^63^. Equal amounts of total protein extracts were analysed by SDS-PAGE (Life Technologies) and western blotting. Proteins were blotted onto 0.45 μm Immobilon-FL PVDF (Merck Millipore) and probed using the indicated antibodies. Blots were visualised using the Odyssey system (LI-COR Biosciences) and bands were quantified for integrated density after background subtraction using ImageJ version 2.0 software (NIH, https://imagej.nih.gov/ij/). Values were normalised to the intensity of the loading control band for each sample.

### Immunofluorescence confocal microscopy

AAT-CHO K1 cells seeded on poly-D-lysine coated coverslips were washed in PBS and fixed in 4% paraformaldehyde/PBS for 30 min at room temperature (RT). Cells were then permeabilised with 0.1% Triton-X/PBS for 5 min and blocked in 3% BSA/10% Normal Goat Serum/PBS for 30 min at RT. Coverslips were incubated with selected primary antibodies overnight at 4 °C, washed with PBS, and treated with the relevant secondary antibodies for 45–60 min at RT, followed by PBS washing and mounting in ProLong Gold with DAPI (Molecular Probes, Invitrogen). Coverslips were cured overnight at RT prior to confocal imaging. Cells were imaged on a Leica TCS SP8 MP OPO confocal microscope (Leica Microsystems) using a HC Plan-Apo CS2 63 × /1.4 N.A. oil immersion objective (Leica Microsystems). Images were acquired sequentially using the LAS-AF acquisition software (Leica Microsystems). All images were then processed using ImageJ version 2.0 and figures compiled using Adobe Illustrator CS6.

### Calcium imaging

Immediately before imaging, AAT-CHO K1 transfected cells on coverslips were washed in Hank’s Balanced Salt Solution (HBBS)^64^ without phenol red (Life Technologies) containing 2 mM calcium (Ca^2+^-HBBS) and supplemented with 20 mM HEPES (Life Technologies). Dynamic measurements of intracellular Ca^2+^ were performed on cells loaded with 4 μM fluorescent Ca^2+^-sensitive probe Fura2-AM (Life Technologies) in Ca^2+^-HBBS containing 0.1% Pluronic F-127 (PF127, Sigma-Aldrich). The PF127 enables high dye-loading efficiency, while preserving cell viability and also expedites repetitive measurements^65^. This treatment was followed by incubation at RT for 40 min. Cells were then washed 3x and bathed in imaging buffer (Ca^2+^-free HBBS, 20 mM HEPES) for recording at RT. Cells were imaged over 7–10 min before and after application of thapsigargin (TG) to a final concentration of 1 μM (Life Technologies) followed by 2 mM Ca^2+^ to evoke store-operated Ca^2+^-entry. Imaging was performed on an Olympus IX71 (Olympus Corporation) inverted epifluorescence microscope using a 20X fluorite/0.45 N.A. air objective lens (Nikon Instruments Inc.). Time-lapse images were acquired using a Hamamatsu C10600–10B CCD camera (Hamamatsu Inc, Imaging Systems) and recorded using Simple PCI version6.6 software (Hamamatsu Inc, Imaging Systems). The light source was provided by a metal halide arc lamp passing through a computer-controlled filter wheel (Prior Scientific). Ca^2+^-levels were assessed by measuring the changes in ratiometric fluorescence (excitation 340 nm/380 nm ± 10 nm, emission 510 nm), every 3 s with exposure of 300 ms. Image processing was performed using ImageJ version 2.0 (NIH) applied to raw non-saturated images and analysed using MATLAB R2015 version 8.5.0.197613 (MathWorks, Inc.). Intracellular calcium changes were expressed as means ± SEM (n = 11 independent experiments, at least 30 cells selected/genotype/experiment).

### Statistical analyses

The parameters measured were basal Ca^2+^, the peak amplitude of the response to TG and the peak amplitude of the calcium transient evoked by the application of calcium ions to TG-treated cells after 7–10 min in Ca^2+^-free medium (SOCE). The fluorescence ratio cannot be negative and therefore cannot be Normally distributed. To simplify analysis, the measurement unit we used (*L*) was the log_10_ of the fluorescence ratio for all cells measured in each experiment. All cells on the coverslip were analysed using our own Python script, which identifies cells in the acquired image using the Difference of Gaussians function provided in the Scipy package. This reduces the danger of bias introduced by the experimenter selecting cells for analysis.

### ANOVAs

We applied one-way independent-measures ANOVAs to the measurements, with STET as the measurement variable and genotype as the only factor.

## Results

### Alpha1-antitrypsin (AAT) is expressed in the AAT-expressing CHO K1 stable cell lines

**Figure 1:**
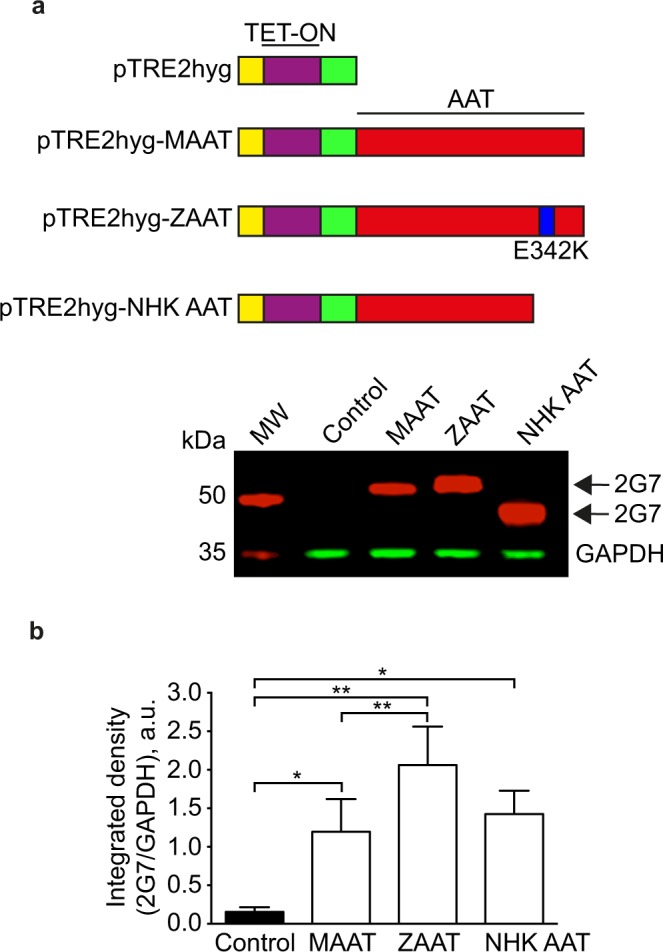
Characterisation of human alpha-1-antitrypsin (AAT) protein in CHO K1 cell lines expressing human wild type and disease variants of AAT. **(a)** Cartoon representation of the doxycycline inducible pTRE2hyg constructs encoding for human AAT proteins expression used in generating the stably AAT expressing CHO K1 cells line^25^. Single amino acid substitution from Glutamate (Glu) to Lysine (Lys) in the AAT sequence at position 342 resulted in the polymerogenic ZAAT (E342K) mutant (blue, 395 aa) that is retained in the ER. Frameshift mutation in the AAT sequence generating a premature stop codon at amino acid position 334 resulted in the NHK AAT mutant. Unlike the ZAAT, the NHK AAT mutant is cleared from the ER through the ER-associated degradation (ERAD) process. Human AAT protein expression in stably AAT expressing CHO K1 and control cells (non-AAT expressing) was evaluated by western blotting using an anti-antitrypsin 2G7 antibody that recognises both monomer and polymer forms of AAT and GAPDH as loading control. Arrows indicate the non-polymerogenic wild type MAAT and polymerogenic disease variant ZAAT proteins (~52 kDa)^86^ and the truncated form of the AAT protein, NHK AAT variant (~45 kDa)^67^. Full image of the blot is included in this paper as Supplemental Fig. 1. **(b)** Densitometry quantification of AAT detection in CHO K1 cells lines, expressed relative to the level of GAPDH. Data points plotted are means ± SEM. *P < 0.05; **P < 0.01 (n = 8 independent experiments). Controls are CHO K1 cells transfected with empty plasmids and otherwise treated in the same way as the AAT lines.

CHOK1 stable cell lines expressing tetracycline-regulated empty or AAT plasmids (Fig. 1a, top panel) were induced using doxycycline as described in the Materials & Methods section for AAT protein translation (n = 8). Using SDS-PAGE and western immunoblotting we confirmed that AAT was only expressed in induced cells and that the expected molecular size value of AAT (~52 kDa) was obtained from extracts of induced cells (Fig. 1a, lower panel, Supplemental Fig. 1). Quantification by densitometry of the immunoblotting showed that cells expressing wild type AAT (MAAT) yielded significantly lower levels of AAT compared to those expressing the disease variants AAT (ZAAT and NHK AAT) (Fig. 1b). The higher intracellular levels observed for ZAAT agreed with previously reported studies showing that the ZAAT variant is retained in the endoplasmic reticulum (ER) of the cells as AAT-protein polymers^25,66^. The NHK AAT variant resolved as a lower molecular size protein (~45 kDa), consistent with a previous finding that this variant formed a misfolded truncated AAT-protein^67^, although it can be cleared through the ERAD system^67–69^.

### Alpha1-antitrypsin (AAT) forms polymers in the endoplasmic reticulum of the AAT-expressing CHO K1 stable cell lines

**Figure 2:**
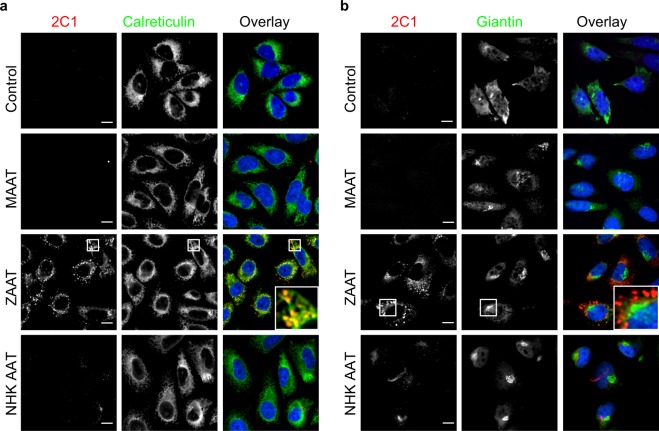
Subcellular localisation of human AAT in the stably AAT expressing CHO K1 cell lines. (**a**,**b**) Representative confocal images displaying control and CHO K1 cells stably expressing wild type MAAT, disease variant ZAAT and a truncated disease variant NHK AAT upon exposure to 1 μg/ml Doxycycline for 48 h, fixed in 4% paraformaldehyde (PFA) followed by fluorescence immunostaining. Cells were counterstained using DAPI nuclear stain. Insets show enlarged images of the association between expressed AAT and either an endoplasmic reticulum (ER) marker calreticulin (**a**) or a Golgi marker giantin (**b**). Scale bar represents 10 μm (n = 3 independent experiments). Controls are CHO K1 cells transfected with empty plasmids and otherwise treated in the same way as the AAT lines. Our results show AAT polymer formation in the endoplasmic reticulum of the AAT-expressing stable CHO K1 cells lines expressing the ZAAT and NHK AAT variants.

We used immunofluorescence cytochemistry to probe the subcellular localisation of the AAT-protein and visualise the retention of AAT-protein polymers in the stably AAT-expressing CHO K1 cells using anti-polymer 2C1 antibody (Fig. 2a,b). Control cells expressing empty and wild type (MAAT) plasmids failed to detect intracellular AAT-polymers (n = 3, Fig. 2a). Similarly, cells expressing NHK AAT showed barely discernible anti-polymer 2C1 antibody staining, whereas the cells expressing ZAAT stained positively confirming that the polymer is expressed (Fig. 2a). To determine whether the AAT-polymers were retained in the ER, or moved to the Golgi en route to clearance, colocalisation studies were undertaken using calreticulin (Fig. 2a) and giantin (Fig. 2b) antibodies as ER and Golgi markers respectively. The 2C1-positive ZAAT-polymers appeared to localise to a degree, albeit not strongly with the ER marker, calreticulin (Fig. 2a) but not giantin (Fig. 2b). All the cells displayed structurally intact and healthy ER. Taken together, our observations support the previous findings that disease variant ZAAT formed AAT-polymers which are trapped in the ER of the CHO K1 cells^25^.

### Fura2-AM Ca^2+^-imaging of CHO K1 stable cell lines expressing wild type (MAAT) and mutant AAT (ZAAT, NHK AAT): measurements of basal intracellular Ca^2+^, responses to thapsigargin and store operated calcium entry

**Figure 3:**
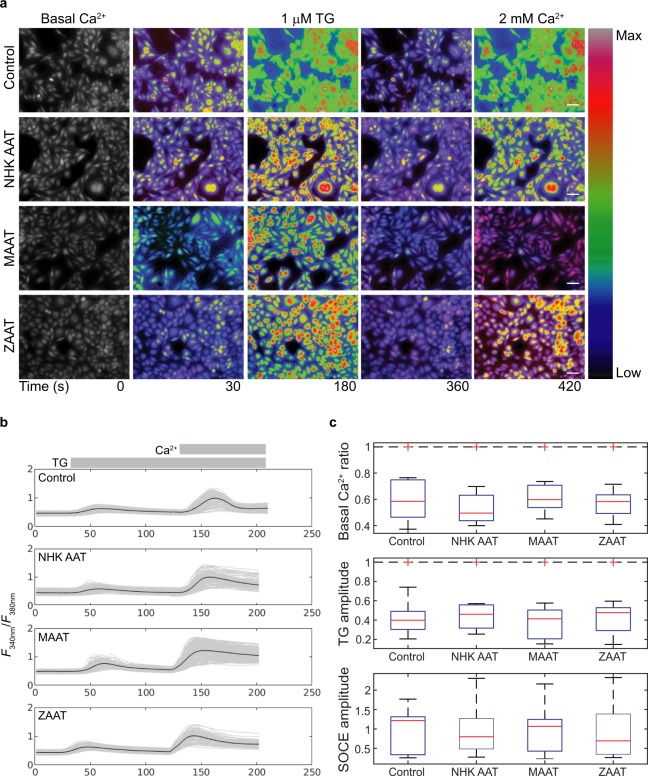
Time-course of changes in intracellular calcium signaling in CHO K1 cell lines stably expressing WT and disease variants of human alpha-1-antitrypsin. (**a**) Fluorescence images of Fura2-AM stained AAT-stably expressing CHO K1 cells (*F*_340 nm_/*F*_380 nm_) in grayscale (t = 0 s) and pseudo-coloured showing intracellular calcium at rest, in calcium free, at peak response to 1 μM thapsigargin (TG); a blocker of the ER calcium ATPase, and finally after restoration of normal extracellular calcium by exposure to 2 mM CaCl_2_, which reveals store-operated calcium-entry (SOCE). Scale bar represents 20 μm. (**b**) Representative traces of calcium measurements obtained at 3 s intervals during exposure to Ca^2+^-free medium, addition of TG and the restoration of external calcium (2 mM CaCl_2_) for control cells and AAT-expressing cells. (**c**) Boxplots summarising the basal intracellular calcium levels (top panel), calcium levels following TG exposure (middle panel)) and following restoration of external calcium (bottom panel) for control cells and 3 genotypes MAAT, ZAAT, NHK AAT (n = 11 independent experiments; 30 cells/genotype/experiment). Controls are CHO K1 cells transfected with empty plasmids and otherwise treated in the same way as the AAT lines.

Polymer retention in the ER has previously been shown to result in ER stress and endoplasmic reticulum overload response (EOR)^25^, which might conceivably affect calcium stores^70^. We therefore used fura2 based calcium imaging on AAT CHO K1 cells expressing wild type (MAAT) and mutant (ZAAT and NHK AAT) forms of AAT. Basal calcium, the response to thapsirgargin (TG) and store-operated Ca^2+^-entry were all measured (Fig. 3). Representative images of AAT CHO K1 cells loaded with the fura2-AM dye show the intracellular Ca^2+^-levels during recordings (Fig. 3a). Baseline Ca^2+^ measurements were obtained as well as a transient elevation of Ca^2+^-levels upon the addition of TG addition in calcium-free bathing medium. This is a well-reported observation from many cell types, which reflects the emptying of internal stores caused by the inhibition of SERCA pumps by TG. The block of SERCA unmasks one aspect of the dynamics of store-control, with the area under the TG-evoked transient being proportional to the volume of the SERCA-regulated intracellular stores and the amplitude reflecting the rate of calcium efflux. In our experiments (Fig. 3b), the amplitude of the TG transient did not differ between the cell types tested (1-way ANOVA, F(3) = 0.1, P = 0.95). Following the TG-evoked transient, we then used the standard method of re-applying calcium in the bathing medium to uncover SOCE. The amplitude of this response is assumed to be proportional to the permeability of the plasma membrane which had been increased in response to store emptying but had been concealed by the absence of calcium in the bathing medium. The amplitude of this SOCE transient did not differ between the cell types studied (1-way ANOVA, F(3) = 0.01, P = 0.998). The basal levels of free Ca^2+^ was also similar between the cell types (1-way ANOVA, F(3) = 0.1, P = 0.96), (Fig. 3c). Although (as illustrated) the calcium transients compared between cell lines exhibited different dynamics in any one experiment, these dynamics did not vary consistently between experiments, suggesting that they are attributable to within-experiment sources of variability such as the way in which the salines were added manually to the experimental chamber.

## Discussion

In this study, we investigated the effect of α_1_-antitrypsin (AAT) deficiency on calcium (Ca^2+^) signalling in CHO K1 cell lines heterologously expressing wild type AAT (MAAT) and AAT polymer forming (ZAAT) and the truncated Null HongKong (NHK AAT) mutants. The CHO K1 cell model system was convenient because of the absence of endogenous AAT^38^, thereby enabling direct evaluation of any impact of introduced AAT on Ca^2+^-dynamics^71^. Previous work from our group provided biochemical understanding of AAT behaviour in the CHO K1 cells^25^. For example, we showed that AAT production could be induced in our transformed CHO K1 cell lines and the AAT proteins either accumulated as polymers in the ER (ZAAT) or were trafficked out of the ER (NHK AAT). The NHK mutant is truncated and is degraded by ERAD. Our current findings accord with previous reports on AAT subcellular expression and localisation^3,25,72^. Microscopy evaluation of the CHO K1 cells showed morphologically intact cells, supporting the view that results could not be attributed to unhealthy cells.

In our experiments, we applied HBBS to which calcium had not been added but did not add a calcium buffer. We therefore cannot exclude the possibility that there remained some residual calcium ions in the saline that might contribute to the transient evoked by thapsigargin, reducing the accuracy of taking the amplitude of this transient as a measure of the volume of intracellular stores. However, if significant levels of calcium were available to the cell from the extracellular saline a store-operated entry would arise, for which we saw no evidence. In addition, the same conditions were applied to all cell lines, and yet no differences emerged between them.

We determined whether the retention of polymers of ZAAT or the truncated NHK AAT expressed in CHO-K1 cells were accompanied by changes in intracellular calcium signalling. We measured baseline free Ca^2+^ and the amplitudes of the TG-induced and SOCE-induced calcium transients in cell lines expressing the disease mutations and in control cell lines expressing wild type AAT (MAAT). An analysis-of-variance approach found no significant differences for these comparisons. To interpret the findings, we assume that the release of Ca^2+^ can be modelled as the result of a flux between three compartments, notably the intracellular Ca^2+^ stores, the cytosol, and the exterior of the cell. It is generally assumed that the TG transient is caused by a block of SERCA, revealing an otherwise counterbalanced leak of Ca^2+^ into the cytosol. The down-sweep of the transient is presumed to be due to clearance of the released Ca^2+^ out of the cell, mostly through passive leak since the experiment is performed in calcium-free medium.

In this simple model, the amplitude of the TG transient will be proportional to the number of Ca^2+^ ions in the intracellular stores. Given these assumptions, the data suggest that retention of polymers in this cell model does not influence intracellular stores. However, we cannot exclude the possibility that the mutations may have effected more subtle changes in calcium handling. Analysing the effects of distinct subpopulations of SH-SY5Y cells on the rates of the rise and fall in the TG response has been used to reveal subtle differences in store handling^73,74^. However, our use of an unstirred chamber meant that such analysis of the rates of rise and fall in the TG and SOCE responses would not have been meaningful. Furthermore, we tested only one concentration of TG, which would probably be around the maximal response. Measuring the amplitude of TG transients and SOCE in response to different concentrations of TG might have uncovered more subtle differences, since the sensitivity of the TG response can be affected by the degree of store filling^75,76^. Further, we have not tested for more subtle differences in calcium signalling such as changes in the spatial pattern of calcium signals such as sparks and local oscillations. This limits the scope of our conclusion, that the AAT mutations do not affect the cell’s ability to handle calcium signalling, to the level of gross changes in calcium signalling and forbids us to preclude the importance of more subtle changes in calcium handling that, though subtle, might nonetheless have important consequences in cell survival.

Retention of intracellular misfolded proteins can cause ER calcium release, which in turn can activate the inflammatory NF-κB pathway as part of the cellular stress response^77–81^. Elevated intracellular calcium beyond the physiological levels in the cells is well known to cause toxicity. This may contribute to Alzheimer’s Disease (AD), a severe type of neurodegenerative disease resulting from accumulation in the brain of harmful amyloid polymers^82,83^. Amyloid oligomers lead to Ca^2+^-signalling dysfunction in neurons, leading in turn to the learning and memory deficits in AD^84^. Patients with cystic fibrosis showed that stimulation of NF-κatients with cystic fibrelial cells is associated with a rise in intracellular calcium levels^85^. Studies on wild type and mutant neuroserpin, the latter being associated with FENIB, another serpinopathy, suggested a possible Ca^2+^- signalling pathway linking the activation of NF-κB with the accumulation of ER polymers. Investigation of a PC12-based model of FENIB showed increased Ca^2+^ release in response to TG in PC12 cells^60^. However, our findings in a CHO K1 cell model of AAT failed to detect a similar response.

The ZAAT and NHK AAT mutations have different effects on AAT processing and structure. The ZAAT mutants formed AAT polymers that accumulate intracellularly^12,26,68^ whereas the NHK AAT mutants formed misfolded protein that can then be removed by the cellular protein degradation system (ERAD)^68,69^. There are different possible explanations for this. One is that the presence of the polymer *per se*, rather than its accumulation, leads to cell damage. Another is that the cell responds to the presence of the polymer with a protective mechanism that may include increasing the volume of the ER in an attempt to increase secretion. In this model, a part of the secretory response downstream from ER enlargement fails in the case of ZAAT, thereby resulting in polymer accumulation. Another possible explanation is that although NHK AAT has a lower propensity to form accumulated aggregates due to the misfolded protein being cleared through ERAD, the levels of expression obtained in our cells may be so high as to mask this difference.

Although it may be relevant in the case of FENIB, we failed to find evidence for an involvement of intracellular Ca^2+^ signalling in serpin-accumulation in the case of AAT with respect to the ZAAT and NHK AAT mutants. Thus, until evidence can be obtained for an involvement of Ca^2+^ in AAT deficiency, therapeutic avenues other than those involving manipulation of aspects of calcium signalling should be explored with the aim of slowing or preventing the development of the disease.

## Supplementary information

Supplemental information

## Data Availability

The data that support the findings in this study are available from the corresponding author, upon reasonable request.
