# Supplementary material for: Calcium signalling in mammalian cell lines expressing wild type and mutant human α1-Antitrypsin
Source: Sci Rep. 2019 Nov 21;9:17293. doi: 10.1038/s41598-019-53535-1 (PMC6872872; doi:10.1038/s41598-019-53535-1)

Supplemental Information

Calcium signalling in mammalian cell lines expressing wild type and mutant human α1-Antitrypsin

Nancy T. Malintan[1,2+], Steven D. Buckingham[1+], David A. Lomas[1] and David B. Sattelle[1*]

[1]Centre for Respiratory Biology, UCL Respiratory, Rayne Building, University College London, 5 University Street, London WC1E 6JF, UK
[2]UCL Queen Square Institute of Neurology, London WC1N 3BG, UK

[+]These authors contributed equally to the project

***Corresponding author:**
Prof. David B. Sattelle
Centre for Respiratory Biology,
UCL Respiratory,
Rayne Building,
University College London,
5 University Street,
London WC1E 6BT, UK
Email: d.sattelle@ucl.ac.uk
Tel: 44 (0) 203 108 7749

Supplemental information figure legend

Supplement figure 1

Representative western immunoblotting showing human AAT expression in stably AAT CHO K1 and control cells (non-AAT expressing). Recombinant purified MAAT protein and MAAT polymer produced from heat exposure[1] were used for comparison. Expression of AAT was evaluated by western immunoblotting in AAT CHO K1 cells lysate and culture media using anti-antitrypsin 2G7 antibody that recognises both monomer and polymer forms of AAT and GAPDH as loading control. Blots were visualised at low (left panel) and high (right panel) exposure levels on the Li-Cor Odyssey scanner followed by image compilation using Adobe Illustrator CS6 for presentation. All blots were results from independent experiments (n = 8 independent western blotting experiments were performed). Detection of AAT in MAAT cell culture media show this protein undergo intracellular processing and secreted. We attributed the detection of ZAAT in the cell media is due to cell death, and minimal as most of the protein is retained intracellularly, shown by the faint AAT signal. The truncated form of AAT protein, NHK AAT is indicated by a lower molecular size and degraded via ERAD, consistent with lack of secretion[2] into the culture media.

References

1 Irving, J. A., Haq, I., Dickens, J. A., Faull, S. V. & Lomas, D. A. Altered native stability is the dominant basis for susceptibility of alpha1-antitrypsin mutants to polymerization. *Biochemical Journal* **460**, 103-115, doi:10.1042/bj20131650 (2014).

2 Sifers, R. N., Brashears-Macatee, S., Kidd, V. J., Muensch, H. & Woo, S. L. A frameshift mutation results in a truncated alpha 1-antitrypsin that is retained within the rough endoplasmic reticulum. *The Journal of biological chemistry* **263**, 7330-7335 (1988).

Supplement Figure 1

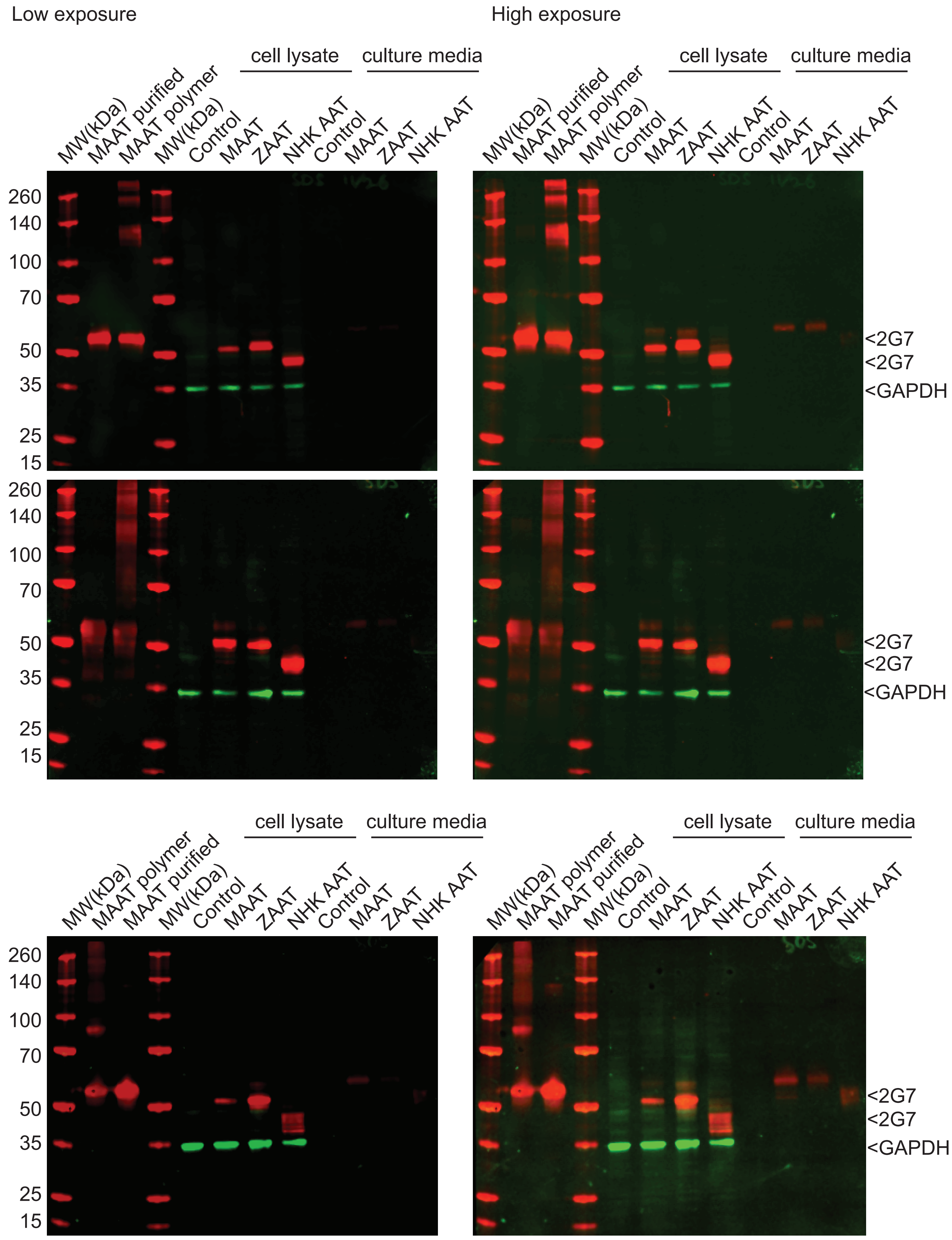

Supplement: Supplementary file 1 — Supplemental information [file 41598_2019_53535_MOESM1_ESM.pdf]
